# Serum Asymmetric Dimethylarginine, Nitrate, Vitamin B_**12**_, and Homocysteine Levels in Individuals with Pulmonary Embolism

**DOI:** 10.1155/2011/215057

**Published:** 2011-06-22

**Authors:** Murat Altuntaş, Figen Atalay, Murat Can, Remzi Altın, Meltem Tor

**Affiliations:** ^1^Department of Pulmonary Medicine, Faculty of Medicine, Zonguldak Karaelmas University, Zonguldak 67600, Turkey; ^2^Department of Biochemistry, Faculty of Medicine, Zonguldak Karaelmas University, Zonguldak 67600, Turkey

## Abstract

We aimed to analyze the pre- and posttreatment serum asymmetric dimethylarginine (ADMA), nitrate (NO_3_), vitamin B_12_ and homocysteine levels in pulmonary embolism (PTE) patients and to determine the prognostic value of these variables in predicting chronic thromboembolic pulmonary hypertension (CTEPH). This study was conducted in 64 patients. The patients were classified into the two groups: patients with normal pulmonary artery pressure (PAP) (group I) and patients with high PAP with persistent lung perfusion defects or who died at the end of 3 months of therapy (group II). We found statistically significant differences between two groups with respect to the partial oxygen pressure, the oxygen saturation, and the PAP, but there was no difference between the two groups with respect to the pretreatment ADMA, NO_3_, or homocysteine levels. The vitamin B_12_ levels were higher in group II. The NO_3_ levels increased and the ADMA and vitamin B_12_ levels decreased with treatment in both groups. These results suggest that these parameters are not predictive of the development of CTEPH.

## 1. Introduction

A pulmonary artery embolism is defined as a partial or complete occlusion of a pulmonary arterial branch. Approximately 70% of cases are caused by pelvic or leg thromboses [1, 2]. Precise figures for the incidence of pulmonary embolism (PTE) are not available. The annual incidence of diagnosed venous thromboembolism (VTE) is 150 to 200 cases per 100,000 population [3]. Considering the unknown number of clinically silent embolisms and the nonspecific clinical presentation, the actual disease frequency is underestimated.

 PTE is a severe and potentially fatal disease when the embolism is massive. CTEPH, which develops after the obliteration of the pulmonary vascular bed by repeated and organized PTE, is one of the less frequently seen forms of PTE and is characterized by unexplained dyspnea and a reduction in exercise capacity [4]. CTEPH is defined as symptomatic pulmonary hypertension (mean pulmonary artery pressure (mPAP) >25 mmHg) with persistent lung perfusion defects [5].

 CTEPH is a life threatening and debilitating disease affecting up to 5% of survivors of PTE [6]. The disease is underdiagnosed, and its true prevalence is still unclear. It is characterized by intraluminal thrombus organization and fibrotic stenoses or complete obliteration of the pulmonary arteries. Pulmonary embolism, either as single or recurrent episode(s), is believed to be the initiating event, followed by progressive vascular remodeling [7]. CTEPH is a common variation of PTE [8]. Endothelial dysfunction, attributable to the reduced bioavailability of endogenous vasodilator substances such as NO, is believed to play an important role in the pathogenesis of pulmonary hypertension [9, 10]. NO is synthesized in the endothelium from L-arginine by NO synthase (NOS), which is present as either endothelial NOS (eNOS) or inducible NOS (iNOS), representing important vascular isoforms. The most abundant endogenous NOS inhibitor is ADMA [11]. Homocysteine is also associated with endothelial dysfunction [12]. Homocysteine is a sulfhydryl amino acid derived from the metabolic conversion of methionine. This conversion is dependent on vitamins (folic acid, B_12_, and B_6_) as cofactors or cosubstrates. Severe hyperhomocysteinemia (homocystinuria), due to inherited metabolic defects in homocysteine metabolism, is associated with a very high risk of VTE, and treatment with vitamins is associated with a dramatic decrease in the VTE risk [13]. The results of earlier studies have suggested that excess levels of homocysteine lead to increases in ADMA levels and the impairment of endothelial function [12, 14]. It has been hypothesized that the NO, ADMA, and homocysteine levels may provide information about the likelihood of the development of CTEPH in PTE patients. Skoro-Sajer et al. found that increased ADMA plasma levels were present in patients with CTEPH. ADMA levels were correlated with the severity of pulmonary vascular disease, and they decreased after pulmonary endarterectomy [15].

 The purpose of our study was to analyze the pre- and posttreatment serum ADMA, NO_3_, vitamin B_12_, and homocysteine levels in PTE patients and to evaluate the predictive value of the variables for development of CTEPH. 

## 2. Materials and Methods

### 2.1. Subjects

The subjects of our study were selected among patients who were admitted to the Zonguldak Karaelmas University Hospital Pulmonary Clinic between March 1, 2009 and December 31, 2009. A total of 100 patients who were diagnosed with PTE were included in the study. The diagnosis of pulmonary embolism was confirmed only when computed tomography angiography (CTPA) showed a pulmonary vascular filling defect or when ventilation-perfusion (V/Q) scintigraphy showed at least two segmental defects without ventilation defects. During the diagnosis period, all the patients underwent echocardiography, and the mean PAPs were measured. Posttreatment echocardiograms were obtained in 50 surviving patients at the end of 3 months. The patients who did not experience a decrease in PAP underwent V/Q scintigraphy to determine if CTEPH had developed. 

 Patients receiving drugs affecting the homocysteine level (vitamin B_6_, vitamin B_12_, folic acid, folinic acid, or fenofibrates) or drugs affecting the ADMA level (L-arginine, ACE inhibitors, metformin and thiazolidinediones, estrogens, vitamin D, folic Acid, all-transretinoic acid, or fenofibrates); patients with end-stage liver disease, end-stage renal disease, acute coronary syndrome, severe congestive heart failure, Alzheimer's disease or preeclampsia; patients in hemorrhagic shock were excluded from the study. 

 Echocardiography was performed in all patients during the diagnosis period. Pulmonary hypertension was defined as mPAP >25 mmHg. Patients' ejection fractions (EF) were measured, and the patients with advanced heart failure (EF (%) < 40) were excluded from the study. None of the patients were on anticoagulant therapy for any event at the time of diagnosis. Patients received either low-molecular-weight heparin or enoxaparin at a fixed dose per kilogram of body weight subcutaneously two times per day or unfractionated heparin at an initial bolus dose of 80 IU per kilogram followed by a continuous intravenous infusion at an initial rate of 18 IU per kilogram per hour. The dose was subsequently adjusted so that the activated partial thromboplastin time (aPTT) was two to three times the control value in normal subjects. aPTTs were determined six hours after the start of treatment and whenever a subtherapeutic aPTT was measured after a dose adjustment. Otherwise, the aPTT was tested daily. In each patient, oral anticoagulant therapy was initiated between the first and third days of the initial heparin therapy and was continued for at least three months. The dose was adjusted to achieve an international normalized ratio (INR) of 2.0 to 3.0. Heparin was stopped after 5 days of combined therapy with oral anticoagulant drug when the INR exceeded 2.0. Three of the patients with a diagnosis of acute massive embolism were given heparin and an oral anticoagulant after thrombolytic treatment (tissue plasminogen activator (tPA), 100 mg over 2 hours). 

The patients were informed about the study, and the study protocol was approved by our hospital's ethics committee (date 03/19/2009 and meeting no. 2009/04). 

### 2.2. Samples

At diagnosis and at the end of 3 months of pulmonary embolism therapy, 20 cc antecubital venous blood was drawn from the patients to evaluate biochemical parameters (e.g., the levels of folic acid, vitamin B_12_, homocysteine, ADMA, and NO_3_). 

### 2.3. Laboratory Investigations

Biochemical levels (folic acid, vitamin B_12_) were measured by enzymatic techniques using a DAX 72 autoanalyzer (Bayer Diagnostics Division, Tarrytown, NY, USA). Sera in biochemistry and hemogram tubes were separated by centrifugation in a cooling centrifuge at 3500 ×g for 10 minutes for the ADMA, NO_2,_ and homocysteine analyses. They were aliquoted into Eppendorf tubes and stored at −80° C for ADMA, NO_2_, and homocysteine measurements. Homocysteine was evaluated using an ELX800 plaque reader with an axis shield diagnostic ELISA kit (Dundee, UK), and the results were recorded. Homocysteine levels of 5–15 mg/dl were considered normal. For vitamin B_12_, values of 19–946 pg/mL were considered normal, and for folic acid, values of 4.6–18.7 ng/mL were considered normal. Serum ADMA levels were evaluated with commercial ELISA kits (Immundiagnostik AG, Bensheim, Germany) using an ELX800 ELISA plaque reader instrument. The results were expressed as *μ*mol/L. Because the half-life of NO is very short (10–30 sec.) and measurement is difficult because it is rapidly oxidized and converted into nitrite (NO_2_) and nitrate (NO_3_), total nitrate levels were used as a reflection of NO levels. The total serum nitrate measurement was performed by the cadmium reduction method [16]. 

## 3. Statistical Analysis

Statistical analysis was done using the SPSS USA (version 13.0) program. The compliance of numerical variables to a normal distribution was assessed using the Kolmogorov-Smirnov test. Definitive statistics were expressed as the mean ± standard deviation for normally distributed data and as the number and percentage for categorical variables. Relationships between categorical variables were assessed by the Chi-Square and Fisher's Exact tests. Nonparametric data were compared with the Mann-Whitney *U* test. Comparisons of pre- and posttreatment values were performed using the Wilcoxon test. Linear relationships between two variables were assessed using the Pearson (for parametric data) and Spearman (for nonparametric data) correlation analyses. Results were evaluated using 95% confidence intervals, and *P* < 0.05 was considered statistically significant. 

## 4. Results

Of 100 patients with PTE diagnosed between March 2009 and December 2009, 36 patients discontinued the study during the followup. Therefore, 64 patients were included in the study. Fifteen patients died during treatment period. The baseline patient characteristics and hemodynamic parameters are summarized in Table 1. Of these 64 patients, 59 (59%) were nonsmokers, 39 (39%) were exsmokers, and 2 (2%) were active smokers. V/Q scintigraphy was performed in 10 of the 50 surviving patients with an elevated PAP determined by control echocardiography at the end of three months of treatment. Perfusion defects were detected in 9 (14%) of the 64 patients, and these patients were considered CTEPH candidates. The patients were classified into two groups: patients with a normal PAP (group I) and patients with a high PAP and/or a moderate to high probability based on V/Q scintigraphy or who died before the end of the 3 months of therapy (group II). Heart failure, renal failure, COPD (chronic obstructive pulmonary disease), and prior malignancy were present in 10 (10%), 5 (5%), 25 (25%), and 13 (13%) of patients, respectively. When the two groups were compared, statistically significant differences were detected with respect to the partial oxygen pressure (*P* < 0.001), the oxygen saturation (*P* < 0.001), and the PAP (*P* = 0.008) (Table 1). There was no statistically significant difference between the two groups with respect to comorbidities. Moreover, there were no statistically significant differences between the two groups in terms of pretreatment ADMA, NO_3_, or homocysteine levels (*P* > 0.05). However, vitamin B_12_ levels were higher in group II (patients with high PAP or who died) compared to group I (patients with normal PAP); this difference was statistically significant (*P* = 0.003) (Table 2). The posttreatment ADMA, NO_3_, homocysteine, and vitamin B_12_ levels were evaluated at the end of the 3 months of therapy. We found that after treatment, the NO_3_ levels increased (*P* < 0.001) from initial low levels, and the ADMA (*P* < 0.001) and vitamin B_12_ levels decreased significantly (*P* < 0.006) from initial high levels; the homocysteine levels did not change significantly (Table 3). At the beginning of the treatment period, the patients were classified into four categories according to their arterial pO_2_ levels as normoxic (*n* = 1), mildly hypoxic (*n* = 34), moderately hypoxic (*n* = 27), or severely hypoxic (*n* = 2). When mildly and moderately hypoxic patients were compared to severely hypoxic patients in terms of the pretreatment ADMA, NO_3_, homocysteine, and mPAP levels, only the mPAP was found to be significantly different between the two groups (*P* = 0.009) (Table 4). High serum homocysteine levels were found in 58% of our patients, and the pre- and posttreatment serum homocysteine and ADMA levels were found to be significantly correlated (*r*: 0.300, *P*:  0.016 and *r*: 0.293, *P*: 0.039, resp.). 

## 5. Discussion

The major novel findings of the present study are as follows (1) In the 4th month of treatment, 14% of acute pulmonary embolism patients still had high PAPs and perfusion defects. (2) Initial high ADMA levels decreased after the treatment, whereas NO_3_ levels increased. Additionally, the vitamin B_12_ levels decreased with anticoagulant treatment. (3) Pretreatment hypoxia was found to be a poor prognostic factor for PTE. (4) In the poor prognosis group, the vitamin B_12_ levels were high, but no statistically significant difference was found between the ADMA, NO_3_, and homocysteine levels.

 There has recently been increasing interest in asymmetric dimethylarginine (ADMA) as a marker and potential mediator of endothelial dysfunction in pulmonary vascular disease patients and as a potent competitive inhibitor of NOS [17]. ADMA is derived from the catabolism of proteins containing methylated arginine residues. Higher ADMA concentrations have been measured in many cardiovascular and metabolic diseases, such as coronary artery disease, congestive heart failure, peripheral arterial occlusive disease, hypercholesterolemia, hypertension, and diabetes mellitus [18, 19]. ADMA has not been measured previously in acute pulmonary embolism patients. In our study, there were no statistically significant differences between the two groups with respect to the pretreatment ADMA and NO_3_ levels. In both groups, the NO_3_ levels increased, and the ADMA and vitamin B_12_ levels decreased with treatment. 

 It is not known clearly how the treatment acts on the ADMA and NO levels. It was concluded that these effects might be the results of compensatory mechanisms related to improvement in hypoxia and/or prolongation of period. Changes in the vessel wall and the coagulation system and chronic hypoxia are of value in predicting CTEPH development [20–22]. Fifteen of our patients died during treatment. We observed that 71% of cases in group II had moderate to severe hypoxia. We concluded that persistent high PAP and hypoxemia, compared with pretreatment values, were found to be significant parameters predicting poor prognosis. 

 CTEPH was recently documented to complicate 3.8% of acute pulmonary embolic events [3, 4]. However, there may be many unreported cases, so the actual number of cases may be higher. At the end of three months of treatment and at the 12-month followup, 9% and 5% of acute pulmonary embolism patients, respectively, showed high PAP and persistent perfusion defect in our study. 

 ADMA has been evaluated in several different classes of pulmonary hypertension. Plasma levels were found to be significantly higher in idiopathic pulmonary arterial hypertension patients than in healthy matched controls [23]. In a recent study of 135 patients diagnosed with CTEPH, the plasma ADMA levels were measured at the time of right heart catheterization and was remeasured in patients who underwent pulmonary endarterectomy. The ADMA level was significantly elevated in patients compared with controls [15]. However, in our study, we could not find any significant difference in the plasma levels of NO_3_ and ADMA with respect to mPAP. The variable conclusions of the published studies to date seem to be related to comparing the ADMA levels in the disease state with healthy controls. Similar to our study, other authors have reported decreased levels of ADMA after treatment (endarterectomy). In addition, the measurement method of ADMA may vary. One of the reasons could be the use of the more sensitive high-performance liquid chromatography (HPLC) analysis method by other authors instead of the ELISA method that was used. In recent years, ELISA has been reported to be compatible with HPLC, but many studies have demonstrated that the HPLC method has better sensitivity and selectivity [24]. 

 It has been reported that elevated homocysteine levels are associated with elevated ADMA levels. Both homocysteine and ADMA are thought to mediate their adverse vascular effects by impairing endothelial nitric oxide-dependent functions. Previous studies have shown that serum ADMA levels were positively correlated with serum homocysteine levels, as shown in our study [25, 26]. Our study showed that the pre- and posttreatment serum homocysteine and ADMA levels were significantly correlated. 

There is a relationship among increased plasma homocysteine, folic acid, and vitamin B_12_ levels and premature arterial disease [12, 13]. Böger et al. [12] observed a correlation between plasma ADMA and homocysteine levels in monkeys with hyperhomocysteinemia. Folic acid, vitamin B_6_, and vitamin B_12_ levels are the significant determinants of homocysteine levels [27]. Serum homocysteine levels were high in 58% of our patients. We found high B_12_ vitamin levels in the poor prognosis group, and the serum levels were decreased after treatment.

In conclusion, the lack of a difference in the pretreatment ADMA and NO_3_ levels between patients with normal and high PAPs, together with the significant decrease in the ADMA level and the increase in the NO_3_ level after the treatment, suggests that these parameters are not predictive of the development of CTEPH. 

## Figures and Tables

**Table 1:** Parameters at the beginning of the treatment in Group I and Group II.

	Group I*	Group II**	*P*
	*n* = 40	*n* = 24	
Age	65.7 ± 13.4	70.9 ± 14.1	0.148
Sex (female/male)	20/20	13/11	0.949
Partial oxygen pressure (mmHg)	62.1 ± 7.4	54.1 ± 9.3	<0.001*
Oxygen saturation (%)	92.8 ± 3.2	86.8 ± 6.2	<0.001*
Mean PAP (mmHg)	39.0 ± 9.6	50.8 ± 21.8	0.008*

*Group I : Patients with normal pulmonary artery pressure at the end of three months of treatment.

**Group II : Patients with high pulmonary artery pressure or who had died at the end of three months of treatment.

**Table 2:** Pretreatment ADMA, NO_3_, homocysteine, and vitamin B_12_ levels in patients with normal pulmonary pressure (Group I) and patients with high pulmonary pressure or who died (Group II).

	Group I	Group II	*P*
	*n* = 40	*n* = 24	
ADMA (*μ*mol/L)	0.63 ± 0.19	0.58 ± 0.26	0.399
NO_3_ (*μ*mol/L)	33.8 ± 2.4	33.8 ± 2.1	0.978
Homocysteine (mg/dl)	19.2 ± 8.8	17.1 ± 8.1	0.358
Vitamin B_12_ (pg/dl)	380.5 ± 244.7	649.3 ± 463.9	0.003*

**Table 3:** ADMA, NO_3_, homocysteine, and vitamin B_12_ levels in patients (before and after 3 months of anticoagulant therapy).

	Before therapy	After 3 months of therapy	
	*n* = 50	*n* = 50	
ADMA (*μ*mol/L)	0.64 ± 0.19	0.49 ± 0.16	<0.001*
NO_3_ (*μ*mol/L)	33.7 ± 2.3	41.5 ± 4.4	<0.001*
Homocysteine (mg/dl)	18.4 ± 8.3	19.3 ± 9.9	0.519
Vitamin B_12_ (pg/dl)	443.3 ± 300.7	355.6 ± 215.9	0.006*

**Table 4:** Pretreatment ADMA, NO_3_, homocysteine and mean PAP levels according to the patients' hypoxia levels.

	Level hypoxia	*n*	Mean ± standard deviation	*P*
ADMA (*μ*mol/L)	Mild	34	0.62 ± 0.22	0.709
	Moderate-severe	29	0.60 ± 0.23	

NO_3_ (*μ*mol/L)	Mild	34	33.8 ± 2.1	0.920
	Moderate-severe	29	33.8 ± 2.4	

Homocysteine (mg/dl)	Mild	34	18.6 ± 9.2	0.964
	Moderate-severe	29	18.5 ± 7.9	

Mean PAP (mmHg)	Mild	34	38.1 ± 10.9	0.009*
	Moderate-severe	29	49.3 ± 19.4	
